# Should a matched sibling donor still be considered the primary option for allogeneic hematopoietic cell transplantation in patients over 50 years of age with myelodysplastic syndrome?

**DOI:** 10.1038/s41409-023-01997-3

**Published:** 2023-05-08

**Authors:** Takaaki Konuma, Hidehiro Itonaga, Ken Ishiyama, Noriko Doki, Naoyuki Uchida, Masashi Sawa, Yuta Katayama, Masatsugu Tanaka, Yasunori Ueda, Makoto Onizuka, Shigesaburo Miyakoshi, Yukiyasu Ozawa, Takahiro Fukuda, Ken-ichi Matsuoka, Junji Tanaka, Takafumi Kimura, Tatsuo Ichinohe, Yoshiko Atsuta

**Affiliations:** 1 Department of Hematology/Oncology, The Institute of Medical Science, The University of Tokyo, Tokyo, Japan; 2 Department of Hematology, Nagasaki University Hospital, Nagasaki, Japan; 3 Department of Hematology, Kanazawa University, Kanazawa, Japan; 4 Hematology Division, Tokyo Metropolitan Cancer and Infectious Diseases Center, Komagome Hospital, Tokyo, Japan; 5 Department of Hematology, Toranomon Hospital, Tokyo, Japan; 6 Department of Hematology and Oncology, Anjo Kosei Hospital, Anjo, Japan; 7 Department of Hematology, Hiroshima Red Cross Hospital & Atomic-Bomb Survivors Hospital, Hiroshima, Japan; 8 Department of Hematology, Kanagawa Cancer Center, Yokohama, Japan; 9 Department of Hematology/Oncology and Transfusion and Hemapheresis Center, Kurashiki Central Hospital, Kurashiki, Japan; 10 Department of Hematology and Oncology, Tokai University School of Medicine, Isehara, Japan; 11 Department of Hematology, Tokyo Metropolitan Geriatric Hospital, Tokyo, Japan; 12 Department of Hematology, Japanese Red Cross Nagoya First Hospital, Nagoya, Japan; 13 Hematopoietic Stem Cell Transplantation Division, National Cancer Hospital, Tokyo, Japan; 14 Department of Hematology and Oncology, Okayama University Hospital, Okayama, Japan; 15 Department of Hematology, Tokyo Women’s Medical University, Tokyo, Japan; 16 Preparation Department, Japanese Red Cross Kinki Block Blood Center, Osaka, Japan; 17 Department of Hematology and Oncology, Research Institute for Radiation Biology and Medicine, Hiroshima University, Hiroshima, Japan; 18 Japanese Data Center for Hematopoietic Cell Transplantation, Nagoya, Japan; 19 Department of Registry Science for Transplant and Cellular Therapy, Aichi Medical University School of Medicine, Nagakute, Japan

**Keywords:** Myelodysplastic syndrome, Myelodysplastic syndrome

## Abstract

Human leukocyte antigen (HLA)-matched sibling donors (MSDs) are the preferred choice for allogeneic hematopoietic cell transplantation (HCT). However, as myelodysplastic syndrome (MDS) is most frequently diagnosed in the elderly, MSDs are also likely to be of advanced age. It is unclear whether an MSD should be considered the primary choice for allogeneic HCT in elderly patients with MDS. We retrospectively compared survival and other outcomes in 1787 patients with MDS over 50 years of age and receiving allogeneic HCT between 2014 and 2020, using either MSD (*n* = 214), 8/8 allele-matched unrelated donor (MUD) (*n* = 562), 7/8 allele-MUD (*n* = 334), or unrelated cord blood (UCB) (*n* = 677) in Japan. In multivariate analysis, compared to MSD transplants, the risk of relapse was significantly lower following 8/8MUD transplants (hazard ratio [HR], 0.74; *P* = 0.047), whereas non-relapse mortality was significantly higher following UCB transplants (HR, 1.43; *P* = 0.041). However, donor type did not determine overall survival, disease-free survival, or graft-versus-host disease (GVHD)-free, relapse-free survival, but chronic GVHD-free, relapse-free survival was better after UCB (HR, 0.80; *P* = 0.025) and 8/8MUD (HR, 0.81; *P* = 0.032) compared to MSD transplants. Our study demonstrated that MSDs are not superior to alternative HCT methods, such as 8/8MUD, 7/8MUD, or UCB, in this population.

## Introduction

Allogeneic hematopoietic cell transplantation (HCT) represents the only curative treatment for myelodysplastic syndrome (MDS). While MDS is predominantly a disease of the elderly, recent advances in transplantation techniques, such as safer conditioning regimens and increased availability of unrelated donors, potentially expand indications for allogeneic HCT in elderly patients with MDS [1, 2].

For allogeneic HCT in MDS and other hematological disorders, a human leukocyte antigen (HLA)-matched sibling donor (MSD) is preferred. The Center for International Blood and Marrow Transplant Research (CIBMTR) demonstrated that MSD and 8/8 allele-matched unrelated donor (MUD) transplants showed similar overall survival (OS) and disease-free survival (DFS), whereas 7/8 allele-MUD transplants showed inferior OS and DFS for patients with MDS [3]. However, as MDS is most frequently diagnosed in the elderly, MSDs are also likely to be of advanced age, which can result in a higher frequency of poor graft cell collection [4, 5] and clonal hematopoiesis of indeterminate potential [6, 7]. Consequently, the identification and availability of an MSD can be severely limited for elderly patients with MDS. The increased availability of unrelated donors or HLA mismatched donors could facilitate allogeneic HCT for MDS patients who lack an MSD.

Older donor age is correlated with an increased incidence of graft-versus-host disease (GVHD) and poor survival after unrelated HCT [8–10]. A previous study by Kollman et al. showed that OS, grades II - IV acute GVHD, and chronic GVHD deteriorated with older donor age (18–30 years, 31–45 years, or >45 years) in a stepwise fashion [8]. A recent study by Kollman et al. showed that OS, non-relapse mortality (NRM), and grades II–IV acute GVHD also deteriorated with older donor age (18–32 years, 33–50 years, or >50 years) in a stepwise fashion [10]. However, the optimal threshold of donor age as a prognostic value has been unclear. For elderly patients with MDS (≥50 years), several studies have shown that the use of younger MUDs (<30 or ≤35 years) has better survival rates compared to older MSDs [11, 12]. Given that survival significantly improves after allogeneic HCT from a MUD and unrelated cord blood (UCB) [13–15], it is unclear whether an MSD should be considered the primary choice for allogeneic HCT in elderly patients with MDS. Here, we compare transplant outcomes in patients over 50 years of age with MDS receiving allogeneic HCT between 2014 and 2020, using either MSD, 8/8 allele-MUD, 7/8 allele-MUD, or UCB in Japan.

## Methods

### Patients

Data were obtained from the Transplant Registry Unified Management Program of the Japanese Data Center for Hematopoietic Cell Transplantation [16, 17]. A total of 1787 patients with MDS, who were over the age of 50 and received their first allogeneic HCT between 2014 and 2020 in Japan, were eligible for the analysis. Of these, 214 received transplants from MSDs, 562 received transplants from 8/8 allele-MUDs, 334 received transplants from 7/8 allele-MUDs, and 667 received transplants from UCB. Patients who received HCT from haploidentical or non-sibling-related donors were excluded, as well as those who lacked data on the donor’s age. This study was approved by the adult MDS working group of the Japanese Society for Transplantation and Cellular Therapy and by the institutional review board at the Institute of Medical Science, The University of Tokyo, where this study was conducted (2022-70-0206).

### Objectives and definitions

The primary objective of this retrospective study was to evaluate whether an MSD resulted in superior survival outcomes in older patients with MDS compared to alternative donor types, including 8/8 allele-MUD, 7/8 allele-MUD, and UCB. The secondary objective was to compare the rates of hematopoietic engraftment, incidences of acute and chronic GVHD, relapse, and NRM among the donor types. The degree of HLA disparity was determined at the antigen level for HLA-A, HLA-B, and HLA-DR for MSD and UCB transplants, whereas it was determined at the allele level for HLA-A, HLA-B, HLA-C and HLA-DR for MUD transplants.

The time from HCT to death defined OS, while DFS was defined as the time from HCT to death or recurrence of MDS. GVHD-free, relapse-free survival (GRFS) was defined as the absence of grade III or IV acute GVHD, chronic GVHD requiring systemic therapy, relapse, or death [18]. Chronic GVHD-free, relapse-free survival (CRFS) was defined as the absence of chronic GVHD, relapse, or death [19]. Relapse was defined as the hematological recurrence of MDS, and NRM was defined as death without MDS recurrence after HCT. Neutrophil engraftment was defined as an absolute neutrophil count of 0.5 × 10^9^/L for three consecutive days. Platelet engraftment was defined as a platelet count of 50 × 10^9^/L for three consecutive days without platelet transfusion. Acute and chronic GVHD were graded according to standard criteria [20, 21].

Performance status (PS) [22], HCT-specific comorbidity index (HCT-CI) [23], and conditioning intensity [24] were classified according to previously described criteria. Karyotype risk was determined according to the international prognostic scoring system [25]. For disease risk at HCT, patients with refractory anemia with an excess of blasts-1 (RAEB-1) and RAEB-2 by the World Health Organization (WHO) classification of 2008 [26], or MDS with excess blasts type1 or type 2 (MDS-EB-1 or MDS-EB-2) by the WHO classification of 2016 [27] were classified as high-risk, and others were classified as low-risk.

### Statistical analysis

The baseline patient characteristics were compared using Chi-squared tests for categorical variables and Kruskal-Wallis tests for continuous variables. The correlation between donor and recipient age was tested by the Spearman rank correlation coefficient. The probabilities of OS, DFS, GRFS, and CRFS were estimated using the Kaplan-Meier method, and the groups were compared using the log-rank test. The probabilities of neutrophil and platelet engraftment, acute GVHD and chronic GVHD, relapse, and NRM were calculated using the cumulative incidence method (taking competing risks into account), and the groups were compared using Gray’s test. Death without hematopoietic engraftment or GVHD was a competing event for hematopoietic engraftment or GVHD. Relapse and NRM were considered mutually competing events.

To estimate hazard ratios (HRs) with a 95% confidence interval (CI), multivariate analysis was performed with a Cox proportional hazard model for OS, DFS, GRFS, and CRFS or a Fine and Gray proportional hazard model for neutrophil and platelet recovery, GVHD, relapse, and NRM. The following factors were included in the multivariate model: donor type (MSD vs. 8/8MUD vs. 7/8MUD vs. UCB), age (<60 years vs. ≥60 years), sex (female vs. male), PS (0-1 vs. 2-4), HCT-CI (0–2 vs. ≥3), karyotype (other than poor vs. poor), disease risk at HCT (low-risk vs. high-risk), conditioning regimen (myeloablative conditioning vs. reduced-intensity conditioning), GVHD prophylaxis (with methotrexate vs. without methotrexate), and the use of antithymocyte globulin (ATG) (no vs. yes). Statistical data analyses were carried out using EZR (Saitama Medical Center, Jichi Medical University, Saitama, Japan), a graphical user interface for R 4.2.2 (R Foundation for Statistical Computing, Vienna, Austria) [28]. Statistical significance was defined as a two-tailed *p*-value of less than 0.05.

## Results

### Baseline characteristics

The baseline characteristics of the patients are presented in Table 1. The median recipient age was 58 years (interquartile range [IQR], 54–61 years) for those with MSDs, 62 years (IQR, 57–65 years) for those with 8/8 MUDs, 61 years (IQR, 56–65 years) for those with 7/8 MUDs, and 64 years (IQR, 58–67 years) for those receiving UCB (*P* < 0.001). The proportion of patients with PS 2–4 was higher in MSD and UCB recipients (*P* = 0.018). A higher proportion of 8/8 MUD and 7/8 MUD recipients had a diagnosis to HCT interval of more than 12 months (*P* < 0.001). MSD recipients less frequently received azacytidine prior to HCT (*P* = 0.024). MDS etiology, karyotype risk, and disease risk at HCT did not differ among the four donor groups. The median donor age was 57 years (IQR, 53–60 years) for MSDs, 40 years (IQR, 33–45 years) for 8/8 MUDs, and 40 years (IQR, 32–45 years) for 7/8 MUDs (*P* < 0.001). The proportion of female donors was lower among 8/8 MUD and 7/8 MUD recipients (*P* < 0.001), resulting in a lower proportion of female donors to male recipients in the 8/8 MUD and 7/8 MUD groups (*P* < 0.001). A higher proportion of UCB recipients received ABO-major/bidirectional mismatched HCT (*P* < 0.001), reduced-intensity conditioning (*P* < 0.001), and mycophenolate mofetil-based GVHD prophylaxis (*P* < 0.001). 7/8 MUD recipients were more likely to receive ATG (*P* < 0.001).

**Table 1:** Patient characteristics based on donor type.

Characteristics	MSD	8/8MUD	7/8MMUD	UCB	*P* value
Number of patients	214	562	334	677	
Median recipient age (IQR), years	58 (54–61)	62 (57–65)	61 (56–65)	64 (58–67)	**<0.001**
Recipient age category					**<0.001**
50–59 years	131 (61.2)	196 (34.9)	130 (38.9)	206 (30.4)	
≥60 years	83 (38.8)	366 (65.1)	204 (61.1)	471 (69.6)	
Recipient sex					0.573
Female	70 (32.7)	175 (31.2)	92 (27.5)	205 (30.3)	
Male	144 (67.3)	386 (68.8)	242 (72.5)	472 (69.7)	
Missing	0	1	0	0	
PS					**0.018**
0–1	195 (91.1)	533 (95.0)	315 (94.3)	614 (90.8)	
2–4	19 (8.9)	28 (5.0)	19 (5.7)	62 (9.2)	
Missing	0	1	0	1	
HCT-CI					0.142
0–2	163 (76.5)	418 (74.6)	263 (78.7)	489 (72.2)	
≥3	50 (23.5)	142 (25.4)	71 (21.3)	188 (27.8)	
Missing	1	2	0	0	
Recipient CMV serostatus					0.317
Negative	31 (15.1)	67 (12.1)	46 (14.5)	73 (11.2)	
Positive	174 (84.9)	485 (87.9)	272 (85.5)	581 (88.8)	
Missing	9	10	16	23	
MDS etiology					0.218
De novo	191 (89.3)	483 (85.9)	299 (89.5)	579 (85.7)	
Secondary	23 (10.7)	79 (14.1)	35 (10.5)	97 (14.3)	
Missing	0	0	0	1	
Karyotype					0.165
Good	82 (39.6)	206 (37.3)	126 (38.2)	220 (33.3)	
Intermediate	33 (15.9)	119 (21.6)	55 (16.7)	128 (19.4)	
Poor	92 (44.4)	227 (41.1)	149 (45.2)	312 (47.3)	
Missing	7	10	4	7	
IPSS at diagnosis					0.278
Low	12 (5.8)	30 (5.6)	19 (5.8)	42 (6.5)	
Intermediate-1	58 (28.2)	176 (33.1)	97 (29.7)	161 (25.0)	
Intermediate-2	83 (40.3)	188 (35.4)	124 (37.9)	248 (38.4)	
High	53 (25.7)	137 (25.8)	87 (26.6)	194 (30.1)	
Missing	8	31	7	32	
WHO classification at HCT					0.604
RA, SLD	9 (4.2)	32 (5.7)	15 (4.5)	29 (4.3)	
RARS, RS-SLD, RS-MLD	5 (2.3)	19 (3.4)	12 (3.6)	32 (4.7)	
RCMD, MLD	46 (21.5)	121 (21.5)	64 (19.2)	128 (18.9)	
MDS-U	8 (3.7)	22 (3.9)	21 (6.3)	19 (2.8)	
MDS with isolated del(5q)	0	1 (0.2)	0	3 (0.4)	
RAEB-1, EB-1	68 (31.8)	151 (26.9)	105 (31.4)	194 (28.7)	
RAEB-2, EB-2	75 (35.0)	209 (37.2)	113 (33.8)	265 (39.1)	
Others	2 (0.9)	5 (0.9)	2 (0.6)	6 (0.9)	
Missing	1 (0.5)	2 (0.4)	2 (0.6)	1 (0.1)	
Disease risk at HCT					0.617
Low-risk	68 (32.2)	195 (35.1)	112 (33.9)	213 (31.7)	
High-risk	143 (67.8)	360 (64.9)	218 (66.1)	459 (68.3)	
Missing	3	7	4	5	
Diagnosis to HCT					**<0.001**
<6 months	19 (8.9)	4 (0.7)	1 (0.3)	97 (14.4)	
6–12 months	89 (41.6)	158 (28.2)	79 (23.7)	210 (31.1)	
≥12 months	106 (49.5)	399 (71.1)	254 (76.0)	368 (54.5)	
Missing	0	1	0	2	
Previous treatment of azacitidine					**0.024**
No	126 (58.9)	264 (47.0)	160 (47.9)	342 (50.6)	
Yes	88 (41.1)	298 (53.0)	174 (52.1)	334 (49.4)	
Missing	0	0	0	1	
Previous treatment of chemotherapy					0.732
No	149 (69.6)	414 (73.7)	243 (72.8)	489 (72.3)	
Yes	65 (30.4)	148 (26.3)	91 (27.2)	187 (27.7)	
Missing	0	0	0	1	
Previous treatment of immunosuppression					0.075
No	203 (94.9)	515 (91.6)	317 (94.9)	641 (94.8)	
Yes	11 (5.1)	47 (8.4)	17 (5.1)	35 (5.2)	
Missing	0	0	0	1	
Graft source					**<0.001**
BM	55 (25.7)	465 (82.7)	295 (88.3)	0	
PBSC	159 (74.3)	97 (17.3)	39 (11.7)	0	
CB	0	0	0	677 (100.0)	
Median donor age (IQR), years	57 (53–60)	40 (33–45)	40 (32–45)	0	**<0.001**
Donor sex					
Female	101 (47.2)	152 (27.0)	88 (26.4)	343 (51.0)	**<0.001**
Male	113 (52.8)	410 (73.0)	245 (73.6)	329 (49.0)	
Missing	0	0	1	5	
Sex incompatibility					**<0.001**
Female to male	67 (31.3)	95 (16.9)	66 (19.8)	247 (36.8)	
Others	147 (68.7)	466 (83.1)	267 (80.2)	425 (63.2)	
Missing	0	1	1	5	
ABO incompatibility					**<0.001**
Match/minor mismatch	165 (77.5)	411 (73.4)	231 (69.2)	411 (61.2)	
Major/bidirectional mismatch	48 (22.5)	149 (26.6)	103 (30.8)	261 (38.8)	
Missing	1	2	0	5	
Conditioning regimen					**<0.001**
MAC	140 (65.4)	340 (60.5)	189 (56.6)	346 (51.1)	
RIC	74 (34.6)	222 (39.5)	145 (43.4)	331 (48.9)	
GVHD prophylaxis					**<0.001**
CI + MTX	201 (93.9)	525 (93.6)	311 (93.1)	386 (57.2)	
CI + MMF	9 (4.2)	25 (4.5)	12 (3.6)	255 (37.8)	
Others	4 (1.9)	11 (2.0)	11 (3.3)	34 (5.0)	
Missing	0	1	0	2	
Use of ATG					**<0.001**
No	195 (91.1)	518 (92.2)	241 (72.2)	645 (95.3)	
Yes	19 (8.9)	44 (7.8)	93 (27.8)	32 (4.7)	
Median follow-up for survivors (IQR), months	36 (17–53)	28 (13–50)	28 (12–50)	27 (12–47)	0.058

*IQR* interquartile range, *PS* performance status, *HCT-CI* hematopoietic cell transplantation-specific comorbidity index, *CMV* cytomegalovirus, *MDS* myelodysplastic syndrome, *IPSS* international prognostic scoring system, *WHO* World Health Organization, *HCT* hematopoietic cell transplantation, *RA* refractory anemia, *SLD* single lineage dysplasia, *RARS* refractory anemia with ringed sideroblasts, *RS-SLD* ringed sideroblasts with single lineage dysplasia, *RS-MLD* ringed sideroblasts with multilineage dysplasia, *RCMD* refractory cytopenia with multilineage dysplasia, *MLD* multilineage dysplasia, *MDS-U MDS* unclassifiable, *RAEB* refractory anemia with an excess of blasts, *EB* excess blasts, *BM* bone marrow, *PBSC* peripheral blood stem cell, *CB* cord blood, *MAC* myeloablative conditioning, *RIC* reduced-intensity conditioning, *GVHD* graft-versus-host disease, *CI* calcineurin inhibitor, *MTX* methotrexate, *MMF* Mycophenolate mofetil, *ATG* antithymocyte globulin, *MSD* matched sibling donor, *MUD* matched unrelated donor, *UCB* unrelated cord blood.

The *P* values in bold are statistically significant (<0.05).

### Hematopoietic engraftment

**Fig. 1:**
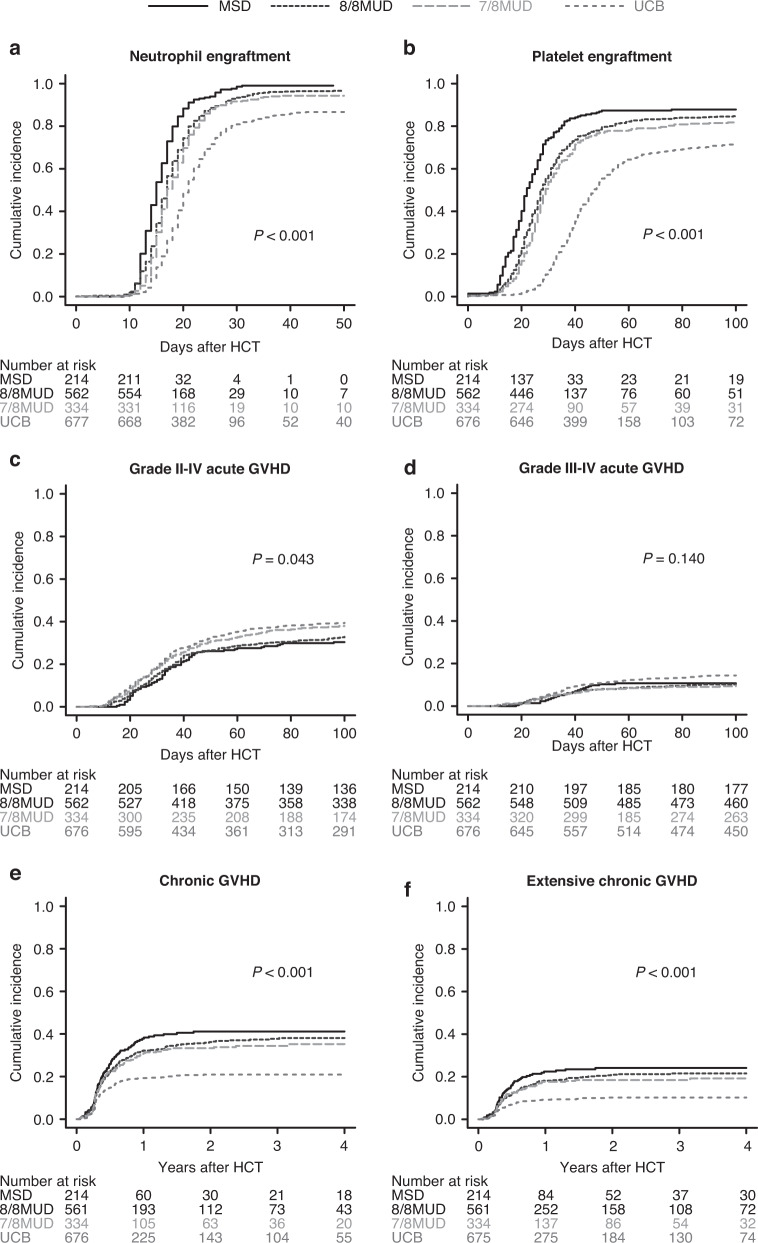
Hematopoietic engraftment and acute and chronic graft-versus-host disease according to donor type. Unadjusted cumulative incidences of neutrophil engraftment (**a**), platelet engraftment (**b**), grades II - IV acute GVHD (**c**), grades III - IV acute GVHD (**d**), chronic GVHD (**e**), and extensive chronic GVHD (**f**) following allogeneic HCT using MSDs, 8/8 MUDs, 7/8 MUDs, or UCB for patients with MDS over 50 years of age.

In univariate analysis, the cumulative incidence of neutrophil engraftment significantly differed among the donor types (*P* < 0.001) (Fig. 1a). In multivariate analysis, compared with MSD recipients, the HR of neutrophil engraftment was significantly lower for 8/8 MUD (HR 0.62 95% CI 0.52–0.74; *P* < 0.001) 7/8 MUD (HR 0.50, 95% CI, 0.41–0.60; *P* < 0.001), and UCB (HR, 0.31, 95% CI, 0.26–0.37; *P*  <  0.001) recipients (Table 2).

**Table 2:** Multivariate analysis of hematopoietic engraftment, and GVHD.

		Neutrophil engraftment		Platelet engraftment		Grades II–IV aGVHD		Grades III–IV aGVHD		cGVHD		Extensive cGVHD	
		HR (95%CI)	*P*	HR (95%CI)	*P*	HR (95%CI)	*P*	HR (95%CI)	*P*	HR (95%CI)	*P*	HR (95%CI)	*P*
Donor													
	MSD	Reference		Reference		Reference		Reference		Reference		Reference	
	8/8MUD	0.62 (0.52–0.74)	**<0.001**	0.67 (0.53–0.84)	**<0.001**	1.14 (0.85–1.51)	0.360	1.08 (0.66–1.75)	0.750	0.86 (0.66–1.12)	0.290	0.84 (0.59–1.19)	0.340
	7/8MUD	0.50 (0.41–0.60)	**<0.001**	0.54 (0.42–0.69)	**<0.001**	1.45 (1.06–1.97)	**0.018**	1.08 (0.62–1.89)	0.770	0.84 (0.90–1.13)	0.270	0.79 (0.53–1.18)	0.270
	UCB	0.31 (0.26–0.37)	**<0.001**	0.31 (0.24–0.38)	**<0.001**	1.44 (1.08–1.91)	**0.012**	1.36 (0.84–2.20)	0.200	0.45 (0.33–0.60)	**<0.001**	0.40 (0.26–0.60)	**<0.001**
Recipient age													
	50–59 years	Reference		Reference		Reference		Reference		Reference		Reference	
	≥ 60 years	1.03 (0.93–1.15)	0.470	1.03 (0.91–1.16)	0.590	0.84 (0.71–0.99)	**0.040**	0.84 (0.63–1.13)	0.260	0.97 (0.80–1.17)	0.800	1.01 (0.78–1.31)	0.900
Recipient sex													
	Female	Reference		Reference		Reference		Reference		Reference		Reference	
	Male	0.91 (0.82–1.01)	0.091	0.80 (0.71–0.90)	**<0.001**	1.03 (0.87–1.22)	0.730	0.97 (0.73–1.30)	0.870	1.11 (0.91–1.34)	0.290	1.26 (0.96–1.65)	0.087
PS													
	0–1	Reference		Reference		Reference		Reference		Reference		Reference	
	2–4	0.83 (0.68–1.02)	0.079	0.61 (0.48–0.78)	**<0.001**	0.99 (0.73–1.35)	1.000	1.07 (0.65–1.77)	0.770	0.76 (0.52–1.13)	0.180	1.03 (0.64–1.65)	0.880
HCT–CI													
	0–2	Reference		Reference		Reference		Reference		Reference		Reference	
	≥3	0.92 (0.83–1.03)	0.180	0.83 (0.73–0.94)	**0.004**	0.88 (0.73–1.06)	0.180	0.99 (0.72–1.37)	0.990	0.89 (0.72–1.10)	0.280	0.88 (0.66–1.18)	0.410
Karyotype													
	Others	Reference		Reference		Reference		Reference		Reference		Reference	
	Poor	1.02 (0.92–1.12)	0.670	1.01 (0.90–1.13)	0.800	1.04 (0.89–1.22)	0.580	0.85 (0.65–1.13)	0.280	0.91 (0.76–1.09)	0.350	0.90 (0.70–1.16)	0.440
Disease risk at HCT													
	Low-risk	Reference		Reference		Reference		Reference		Reference		Reference	
	High-risk	0.98 (0.89–1.09)	0.820	0.99 (0.88–1.11)	0.880	1.01 (0.85–1.20)	0.860	1.02 (0.76–1.36)	0.870	0.93 (0.77–1.12)	0.460	0.88 (0.68–1.13)	0.320
Conditioning regimen													
	MAC	Reference		Reference		Reference		Reference		Reference		Reference	
	RIC	1.07 (0.97–1.19)	0.160	1.10 (0.98–1.23)	0.080	0.90 (0.76–1.06)	0.220	0.79 (0.58–1.05)	0.110	0.81 (0.67–0.98)	**0.033**	0.81 (0.63–1.06)	0.130
GVHD prophylaxis													
	With MTX	Reference		Reference		Reference		Reference		Reference		Reference	
	Without MTX	1.22 (1.06–1.40)	**0.003**	1.00 (0.87–1.16)	0.900	0.99 (0.81–1.22)	0.970	1.06 (0.75–1.50)	0.710	1.19 (0.93–1.54)	0.160	1.04 (0.72–1.52)	0.800
Use of ATG													
	No	Reference		Reference		Reference		Reference		Reference		Reference	
	Yes	1.46 (1.22–1.74)	**<0.001**	1.35 (1.12–1.63)	**0.001**	0.79 (0.59–1.04)	0.100	0.42 (0.21–0.82)	**0.011**	0.83 (0.61–1.13)	0.240	0.79 (0.51–1.21)	0.280

*GVHD* graft-versus-host disease, *MSD* matched sibling donor, *MUD* matched unrelated donor, *UCB* unrelated cord blood, *PS* performance status, *HCT-CI* hematopoietic cell transplantation-specific comorbidity index, *HCT* hematopoietic cell transplantation, *MAC* myeloablative conditioning, *RIC* reduced-intensity conditioning, *GVHD* graft-versus-host disease, *MTX* methotrexate, *ATG* antithymocyte globulin, *HR* hazard ratio, *CI* confidence interval. The *P* values in bold are statistically significant (<0.05).

In univariate analysis, the cumulative incidence of platelet engraftment also significantly differed among the donor types (*P* < 0.001) (Fig. 1b). In multivariate analysis, compared with MSD recipients, the HR of platelet engraftment was also significantly lower in 8/8 MUD (HR, 0.67, 95% CI, 0.53–0.84; *P* < 0.001), 7/8 MUD (HR, 0.54, 95% CI, 0.42–0.69; *P* < 0.001), and UCB (HR, 0.31, 95% CI, 0.24–0.38; *P*  <  0.001) recipients (Table 2).

### GVHD

The cumulative incidences of grades II–IV acute GVHD significantly differed among the donor types (*P* = 0.043) (Fig. 1c), but grades III to IV acute GVHD were not different (*P* = 0.140) (Fig. 1d). In multivariate analysis, the HR of grades II–IV acute GVHD was higher in 7/8 MUD (HR, 1.45, 95% CI, 1.06 to 1.97; *P* = 0.018) and UCB (HR, 1.44, 95% CI, 1.08 to 1.91; *P* = 0.012) recipients compared with MSD recipients (Table 2). However, there was no significant difference in grades III - IV acute GVHD between MSD and either 8/8 MUD, 7/8 MUD, or UCB recipients (Table 2).

In univariate analysis, the cumulative incidences of chronic GVHD (*P* < 0.001) (Fig. 1e) and extensive chronic GVHD (*P* < 0.001) (Fig. 1f) differed among the donor types. In multivariate analysis, the HRs of chronic GVHD (HR, 0.45, 95% CI, 0.33 to 0.60; *P* < 0.001) and extensive chronic GVHD (HR, 0.40, 95% CI, 0.26 to 0.60; *P* < 0.001) were significantly lower in UCB recipients compared with MSD recipients (Table 2).

### Relapse and NRM

**Fig. 2:**
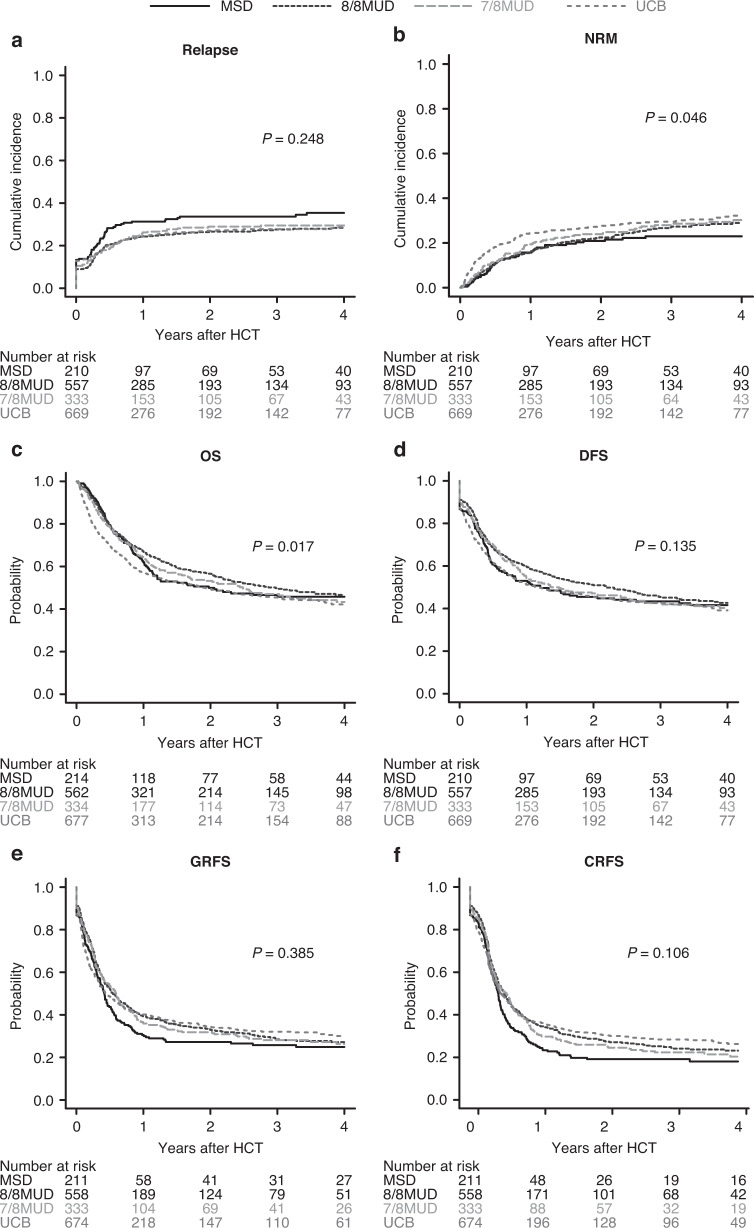
Relapse, non-relapse mortality, and survival outcomes according to donor type. Unadjusted cumulative incidences of relapse (**a**), NRM (**b**), and the unadjusted probabilities of OS (**c**), DFS (**d**), GRFS (**e**), and CRFS (**f**) following allogeneic HCT using MSDs, 8/8 MUDs, 7/8 MUDs, and UCB for patients with MDS over 50 years of age.

The cumulative incidence of relapse did not differ among the donor types in univariate analysis (*P* = 0.248) (Fig. 2a). In multivariate analysis, the HR of relapse was significantly lower in 8/8 MUD recipients compared with MSD recipients (HR, 0.74, 95% CI, 0.56–0.99; *P* = 0.047) (Table 3).

**Table 3:** Multivariate analysis of relapse, NRM, and survival outcomes.

		Relapse		NRM		OS		DFS		GRFS		CRFS	
		HR (95%CI)	*P*	HR (95%CI)	*P*	HR (95%CI)	*P*	HR (95%CI)	*P*	HR (95%CI)	*P*	HR (95%CI)	*P*
Donor													
	MSD	Reference		Reference		Reference		Reference		Reference		Reference	
	8/8MUD	0.74 (0.56–0.99)	**0.047**	1.21 (0.86–1.71)	0.260	0.90 (0.71–1.13)	0.390	0.86 (0.69–1.08)	0.208	0.84 (0.69–1.03)	0.101	0.81 (0.67–0.98)	**0.032**
	7/8MUD	0.74 (0.54–1.01)	0.064	1.38 (0.95–2.01)	0.083	1.00 (0.77–1.29)	0.960	0.94 (0.73–1.20)	0.622	0.93 (0.75–1.15)	0.539	0.88 (0.72–1.09)	0.256
	UCB	0.79 (0.58–1.06)	0.120	1.43 (1.01–2.02)	**0.041**	1.15 (0.91–1.46)	0.230	1.04 (0.82–1.30)	0.728	0.86 (0.70–1.05)	0.152	0.80 (0.65–0.97)	**0.025**
Recipient age													
	50–59 years	Reference		Reference		Reference		Reference		Reference		Reference	
	≥ 60 years	1.45 (1.18–1.78)	**<0.001**	1.01 (0.82–1.24)	0.890	1.24 (1.07–1.44)	**0.003**	1.31 (1.14–1.51)	**<0.001**	1.19 (1.04–1.35)	**0.007**	1.14 (1.01–1.29)	**0.028**
Recipient sex													
	Female	Reference		Reference		Reference		Reference		Reference		Reference	
	Male	1.09 (0.89–1.33)	0.400	1.10 (0.89–1.36)	0.340	1.17 (1.01–1.37)	**0.036**	1.12 (0.96–1.29)	0.127	1.10 (0.96–1.25)	0.145	1.13 (0.99–1.28)	0.055
PS													
	0–1	Reference		Reference		Reference		Reference		Reference		Reference	
	2–4	1.67 (1.26–2.22)	**<0.001**	1.21 (0.85–1.72)	0.270	1.84 (1.47–2.32)	**<0.001**	1.74 (1.39–2.18)	**<0.001**	1.55 (1.26–1.91)	**<0.001**	1.43 (1.16–1.76)	**<0.001**
HCT–CI													
	0–2	Reference		Reference		Reference		Reference		Reference		Reference	
	≥3	1.02 (0.83–1.24)	0.850	1.30 (1.06–1.60)	**0.011**	1.32 (1.13–1.53)	**<0.001**	1.22 (1.05–1.41)	**0.006**	1.09 (0.95–1.24)	0.192	1.11 (0.97–1.26)	0.104
Karyotype													
	Others	Reference		Reference		Reference		Reference		Reference		Reference	
	Poor	2.33 (1.93–2.81)	**<0.001**	0.89 (0.74–1.07)	0.240	1.64 (1.43–1.89)	**<0.001**	1.67 (1.46–1.90)	**<0.001**	1.39 (1.24–1.57)	**<0.001**	1.45 (1.29–1.62)	**<0.001**
Disease risk at HCT													
	Low-risk	Reference		Reference		Reference		Reference		Reference		Reference	
	High-risk	1.14 (0.93–1.38)	0.180	0.96 (0.79–1.17)	0.720	1.03 (0.89–1.19)	0.681	1.06 (0.92–1.22)	0.371	1.06 (0.94–1.20)	0.315	1.08 (0.96–1.22)	0.104
Conditioning regimen													
	MAC	Reference		Reference		Reference		Reference		Reference		Reference	
	RIC	0.82 (0.68–0.99)	**0.041**	0.89 (0.73–1.08)	0.250	0.76 (0.65–0.87)	**<0.001**	0.80 (0.69–0.91)	**0.001**	0.77 (0.68–0.87)	**<0.001**	0.76 (0.68–0.86)	**<0.001**
GVHD prophylaxis													
	With MTX	Reference		Reference		Reference		Reference		Reference		Reference	
	Without MTX	0.69 (0.53–0.90)	**0.008**	1.07 (0.83–1.37)	0.580	0.84 (0.69–1.02)	0.079	0.82 (0.68–0.98)	**0.033**	0.93 (0.79–1.09)	0.386	0.96 (0.82–1.13)	0.658
Use of ATG													
	No	Reference		Reference		Reference		Reference		Reference		Reference	
	Yes	0.96 (0.71–1.30)	0.830	0.72 (0.50–1.02)	0.069	0.75 (0.58–0.97)	**0.031**	0.82 (0.64–1.04)	0.109	0.67 (0.54–0.83)	**<0.001**	0.76 (0.62–0.93)	**0.008**

*NRM* non-relapse mortality, *OS* overall survival, *DFS* disease-free survival, *GRFS* graft-versus-host disease (GVHD)-free, relapse-free survival, *CRFS* chronic GVHD-free relapse-free survival, *MSD* matched sibling donor, *MUD* matched unrelated donor, *UCB* unrelated cord blood, *PS* performance status, *HCT-CI* hematopoietic cell transplantation-specific comorbidity index, *HCT* hematopoietic cell transplantation, *MAC* myeloablative conditioning, *RIC* reduced-intensity conditioning, *GVHD* graft-versus-host disease, *MTX* methotrexate, *ATG* antithymocyte globulin, *HR* hazard ratio, *CI* confidence interval. The *P* values in bold are statistically significant (<0.05).

In univariate analysis, the cumulative incidence of NRM significantly differed among the donor types (*P* = 0.046) (Fig. 2b). In multivariate analysis, the HR of NRM was significantly higher in UCB recipients compared with MSD recipients (HR, 1.43, 95% CI, 1.01–2.02; *P* = 0.041) (Table 3).

### Survival

With a median follow-up of 29 months for survivors in the entire cohort, the probability of OS significantly differed among the donor types (*P* = 0.017) (Fig. 2c). However, the probability of DFS (*P* = 0.135), GRFS (*P* = 0.385), and CRFS (*P* = 0.106) was comparable among the donor types (Fig. 2d–f). In multivariate analysis, there were no significant differences in OS, DFS, or GRFS between MSD and 8/8 MUD, 7/8 MUD, or UCB recipients (Table 3). The HR of CRFS was significantly better in 8/8 MUD (HR, 0.81, 95% CI, 0.67–0.98; *P* = 0.032) and UCB (HR, 0.80, 95% CI, 0.65–0.97; *P* = 0.025) recipients compared with MSD recipients (Table 3).

### Cause of death

The causes of death in each donor type are given in Table 4. The most frequent cause of death was relapse/progression of MDS among each donor type. Relapse/progression and organ failure were more common causes of death after MSD transplants, while infection was a more common cause of death, and GVHD was less common, after UCB transplants. Pulmonary complications and hemorrhage were comparable among the donor types.

**Table 4:** Cause of death according to donor type.

	MSD	8/8MUD	7/8MUD	UCB
Relapse/progression	42 (38.2)	99 (37.6)	57 (36.1)	117 (33.3)
Infection	22 (20.0)	52 (19.8)	33 (20.9)	86 (24.5)
GVHD	10 (9.1)	25 (9.5)	14 (8.9)	21 (6.0)
Pulmonary complication	12 (10.9)	29 (11.0)	18 (11.4)	39 (11.1)
Organ failure	12 (10.9)	23 (8.7)	10 (6.3)	26 (7.4)
Hemorrhage	5 (4.5)	11 (4.2)	8 (5.1)	19 (5.4)
SOS/TMA	2 (1.8)	11 (4.2)	4 (2.5)	16 (4.6)
Secondary cancer	2 (1.8)	8 (3.0)	6 (3.8)	9 (2.6)
Graft failure	2 (1.8)	2 (0.8)	5 (3.2)	10 (2.8)
Exogeneous deaths	1 (0.9)	1 (0.4)	0	1 (0.3)
Unknown	0	2 (0.8)	3 (1.9)	7 (2.0)

*GVHD* graft-versus-host disease, *SOS* sinusoidal obstruction syndrome, *TMA* thrombotic microangiopathy.

### Donor age and outcomes

Because previous studies have shown that older donor age is correlated with an increased incidence of GVHD and poor survival after unrelated HCT [8–10], we investigated whether donor age affects survival and GVHD following MSD, 8/8 MUD, or 7/8 MUD transplants. By the Spearman rank correlation coefficient, donor age was correlated with recipient age among MSD transplantation (*r* = 0.612, *P* < 0.0001), but not in 8/8 MUD (*r* = −0.052, *P* = 0.216) or 7/8 MUD (*r* = 0.115, *P* = 0.034) transplantations (Supplementary Figure 1).

The cumulative incidences of grades III–IV acute GVHD significantly differed among the donor age groups (35 years vs. 35–49 years vs. 40–44 years vs. ≥45 years) only in 8/8 MUD transplants (*P* = 0.024) (Supplementary Fig. 2b), not in MSD or 7/8 MUD transplantations (Supplementary Fig. 2a, c). There was no significant difference in chronic GVHD among the donor age groups in MSD, 8/8 MUD, or 7/8 MUD transplants (Supplementary Fig. 2d–f). Although the probability of OS significantly differed among donor age groups (<50 years vs. 50–54 years vs. 55–59 years vs. ≥60 years) for MSD transplants (*P* = 0.011) (Supplementary Fig. 3a), we did not find an improvement in survival with younger donors (<35 years vs. 35–49 years vs. 40–44 years vs. ≥45 years) in 8/8 MUD or 7/8 MUD transplantations (Supplementary Fig. 3b, c). The probability of GRFS significantly differed among the donor age groups in 8/8 MUD transplants (*P* = 0.004), but not in MSD or 7/8 MUD transplantations (Supplementary Fig. 4).

**Fig. 3:**
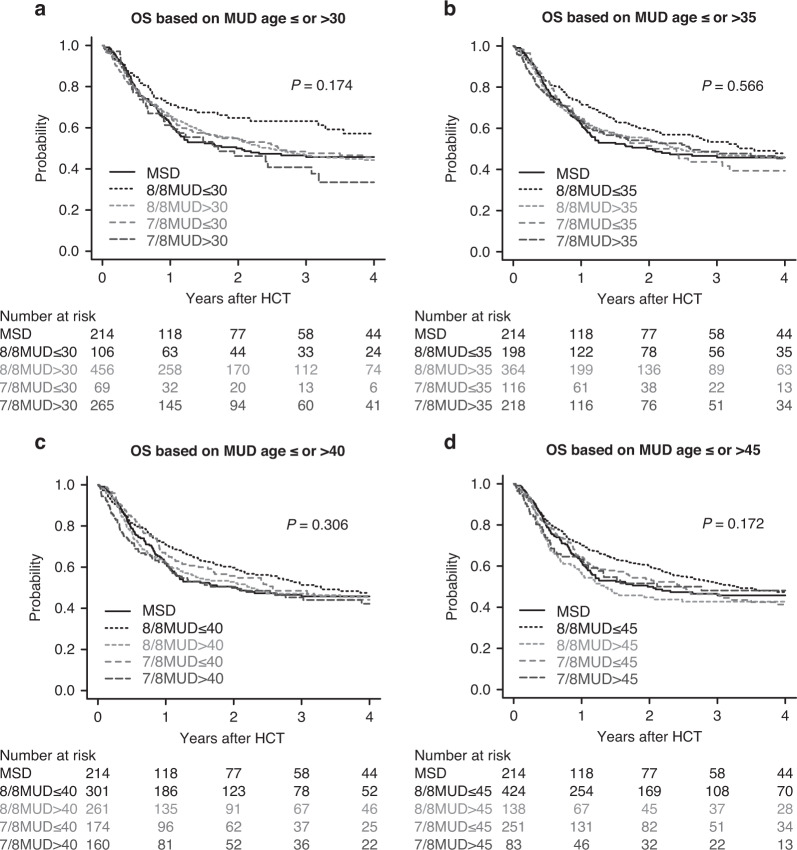
Overall survival based on matched unrelated donor age. Unadjusted probabilities of OS following allogeneic HCT using MSDs, younger or older 8/8 MUDs, and younger or older 7/8 MUDs using different thresholds of donor age among MUDs (30 [**a**], 35 [**b**], 40 [**c**], and 45 [**d**] years) for patients with MDS over 50 years of age.

**Fig. 4:**
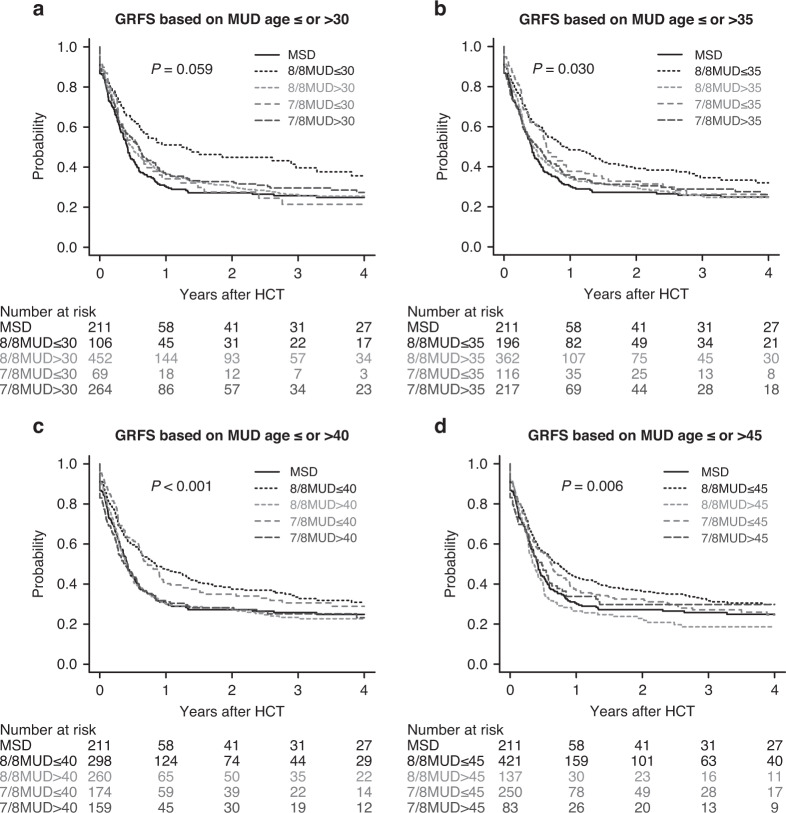
Graft-versus-host disease-free, relapse-free survival based on matched unrelated donor age. Unadjusted probabilities of GRFS following allogeneic HCT using MSDs, younger or older 8/8 MUDs, and younger or older 7/8 MUDs using different thresholds of donor age among MUDs (30 [**a**], 35 [**b**], 40 [**c**], and 45 [**d**] years) for patients with MDS over 50 years of age.

Finally, we also investigated the impact of unrelated donor age on OS and GRFS using different thresholds of unrelated donor age (30, 35, 40, and 45 years), except for the cohort of UCB recipients. Univariate analysis showed that younger 8/8 MUD (≤30 years) recipients had the best OS (Fig. 3), but there was a marginal difference between younger 8/8 MUD (≤30 years) and MSD recipients in multivariate analysis (HR, 0.71, 95% CI, 0.49–1.03; *P* = 0.075) (Supplementary Table 1). In univariate analysis, the probability of GRFS significantly differed among donor types (Fig. 4). In multivariate analysis, the HR of GRFS was significantly better in younger 8/8 MUD recipients compared with MSD recipients, irrespective of thresholds of donor age among 8/8 MUDs (HR, 0.65, 95% CI, 0.48–0.89; *P* = 0.007 for younger 8/8MUD ≤ 30 years, HR, 0.69, 95% CI, 0.54–0.89; *P* = 0.004 for younger 8/8MUD ≤ 35 years, HR, 0.73, 95% CI, 0.59–0.92; *P* = 0.007 for younger 8/8MUD ≤ 40 years, HR, 0.77, 95% CI, 0.63–0.95; *P* = 0.016 for younger 8/8MUD ≤ 45 years) (Supplementary Table 2).

## Discussion

This was a nationwide registry-based study that compared the outcomes of 1787 patients with MDS over 50 years of age receiving allogeneic HCT by MSDs, 8/8 MUDs, 7/8 MUDs, or UCB between 2014 and 2020 in Japan. Our study demonstrated that MSDs were the best in terms of neutrophil and platelet engraftments among the four donor types. The relapse rate was significantly lower following 8/8 MUD transplants, whereas NRM was significantly higher following UCB transplants compared to MSD transplants. However, donor type did not determine OS, DFS, or GRFS. These data suggest that an MSD should not always be considered the primary choice for allogeneic HCT in patients with MDS over 50 years of age.

Previous studies have investigated whether older MSDs or younger MUDs provide better survival outcomes for elderly patients, but conflicting results have emerged [11, 12, 29, 30]. Among leukemia and lymphoma older patients (≥50 years) receiving allogeneic HCT, the CIBMTR showed that OS was significantly better with older (≥50 years) MSDs compared with younger (<50 years) MUDs among recipients with good PS. In contrast, OS was comparable among recipients with poor PS [29]. Focusing on elderly patients with MDS (≥50 years), the European Group for Blood and Marrow Transplantation demonstrated that younger MUDs (<30 years) led to better OS compared to MSDs [11]. This finding was compatible with a recent cohort study of the CIBMTR database showing that relapse rate, DFS, and GRFS were significantly better following younger MUD transplants (≤35 years) than older MSD transplants (≥50 years) [12]. In contrast, given that donor age is usually associated with recipient age in MSD transplants and donor age did not affect OS in 8/8 MUD and 7/8 MUD transplants, our data showed that MUDs do not lead to superior OS compared to MSDs for elderly patients with MDS, irrespective of donor age using different thresholds of unrelated donor age (30, 35, 40, and 45 years). The precise reason for the difference between the previous studies and our cohort is unclear, but the lower incidences of acute and chronic GVHD after MSD and MUD transplantations in our cohort may be a contributing factor [12]. Given that racial differences could affect rates of GVHD in MSD and MUD transplants [31, 32], the relatively homogeneous non-HLA immune genetics among Japanese recipients contributes to the lower incidence of GVHD from MSD and MUD transplantations in our cohort. These data suggested that OS and DFS were comparable after older MSD and MUD transplantations for elderly MDS.

UCB is a suitable alternative donor source for adult patients who lack an HLA-matched related or unrelated donor. Several prior studies have demonstrated similar survival rates between MSD and UCB transplantations for adult patients with hematologic malignancies [30, 33–37], but comparative data for older MSD and UCB transplantations have been limited solely to elderly patients with MDS. Although our prior study showed that MSDs led to better OS compared to UCB for patients over 50 years of age with acute lymphoblastic leukemia, acute myeloid leukemia, and MDS in a Japanese cohort (between 2007 and 2012) [38], our current study (between 2014 and 2020) focusing on elderly patients with MDS demonstrated that UCB did not lead to inferior OS compared to MSDs for elderly patients with MDS. This might be due to the recent improvements in graft failure rates and early NRM after UCB transplants for Japanese adults [15]. As a result, the survival rate was comparable after older MSD and UCB transplantations for elderly patients with MDS.

Older recipient age has also been associated with a higher incidence of chronic GVHD rather than acute GVHD [9]. The lower incidence of chronic GVHD is an attractive advantage of UCB transplantation [33, 34, 39, 40]; UCB could be a useful donor source for elderly patients with MDS. Indeed, our data showed that the incidences of chronic GVHD and extensive chronic GVHD after UCB transplantation were the lowest among the four donor types. Moreover, compared to MSD transplants, CRFS was better after UCB and 8/8 MUD transplants. Therefore, our study suggested that UCB and 8/8 MUD should be preferred over older MSDs in terms of CRFS endpoint, which measures ideal recovery without ongoing morbidity, for elderly patients with MDS.

Our study had several limitations. First, this was a registry-based retrospective study. Treating physicians or institutions might enact alternative donor selection procedures. In addition, data regarding the genetic profile and maintenance therapy were not available in our registry. Second, the use of post-transplant cyclophosphamide as GVHD prophylaxis has resulted in a drastic expansion of haploidentical-related donor transplantations for MDS in recent years [41–43]. However, we could not evaluate haploidentical-related donors because of the small sample size of this population in Japan. Therefore, further studies are warranted to clarify the significance of haploidentical-related donors for elderly patients with MDS. Third, our results should be interpreted with caution when applied to other racial cohorts, because of the frequent use of ATG for GVHD prophylaxis in patients undergoing MUD transplantation in European countries [44], and the different impact of GVHD on outcomes after adult single-unit UCB transplantation in European and Japanese populations [45]. Despite several limitations, the strength of our study was its relatively large number of elderly patients and its focus on MDS to compare efficacy and safety between older MSDs and alternative donors, such as 8/8 MUDs, 7/8 MUDs, or UCB.

In summary, our registry-based study demonstrated that MSDs do not lead to superior OS or DFS compared to alternative HCT from 8/8 MUDs, 7/8 MUDs, or UCB for patients with MDS over 50 years of age. While MSDs were the best in terms of neutrophil and platelet engraftments among the four donor types, an MSD should not always be considered the primary choice for allogeneic HCT in this population.

## Supplementary information

Supplementary information

## Data Availability

The data that support the findings of this study are available from the corresponding author upon reasonable request.
