# Supplementary material for: Should a matched sibling donor still be considered the primary option for allogeneic hematopoietic cell transplantation in patients over 50 years of age with myelodysplastic syndrome?
Source: Bone Marrow Transplant. 2023 May 8;58(8):893–906. doi: 10.1038/s41409-023-01997-3 (PMC10400426; doi:10.1038/s41409-023-01997-3)
Supplement: Supplementary file 1 — Supplementary information [file 41409_2023_1997_MOESM1_ESM.pdf]

Supplementary Figure 1. Correlation between donor age and recipient age in MSD (a), 8/8MUD (b), and 7/8MUD (C) transplantations.

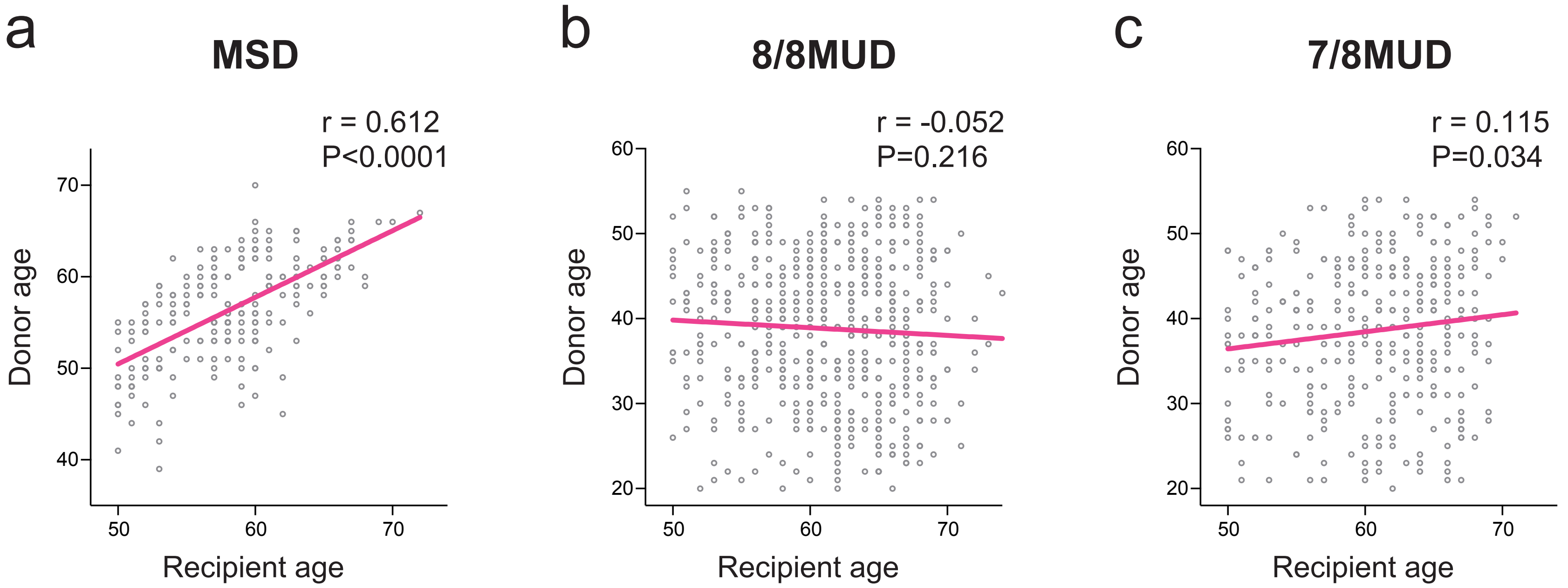

Supplementary Figure 2. Unadjusted cumulative incidences of grades III-IV acute GVHD and chronic GVHD following allogeneic HCT using MSDs (a,d), 8/8 MUDs (b,e), and 7/8 MUDs (c,f) for patients with MDS over 50 years of age according to donor age.

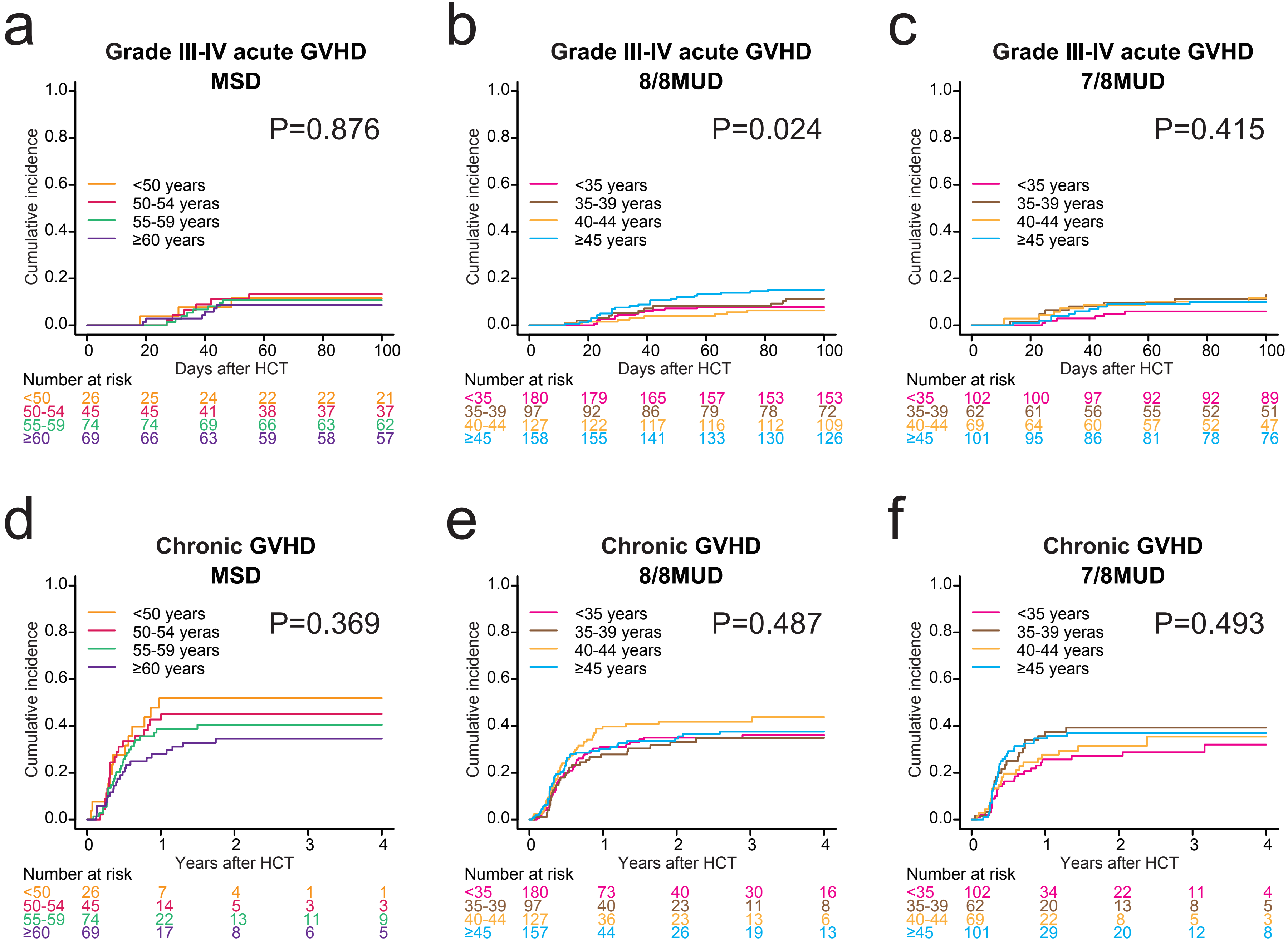

Supplementary Figure 3. Unadjusted probabilities of OS following allogeneic HCT using MSDs (a), 8/8 MUDs (b), and 7/8 MUDs (c) for patients with MDS over 50 years of age according to donor age.

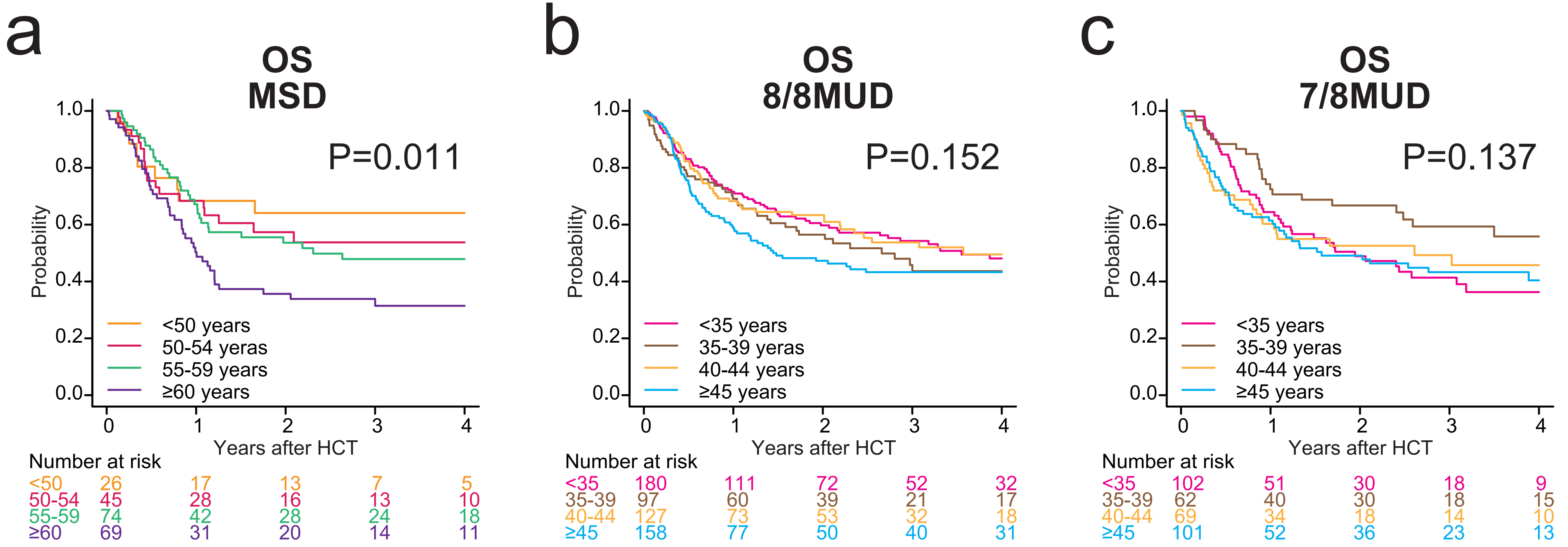

Supplementary Figure 4. Unadjusted probabilities of GRFS following allogeneic HCT using MSDs (a), 8/8 MUDs (b), and 7/8 MUDs (c) for patients with MDS over 50 years of age according to donor age.

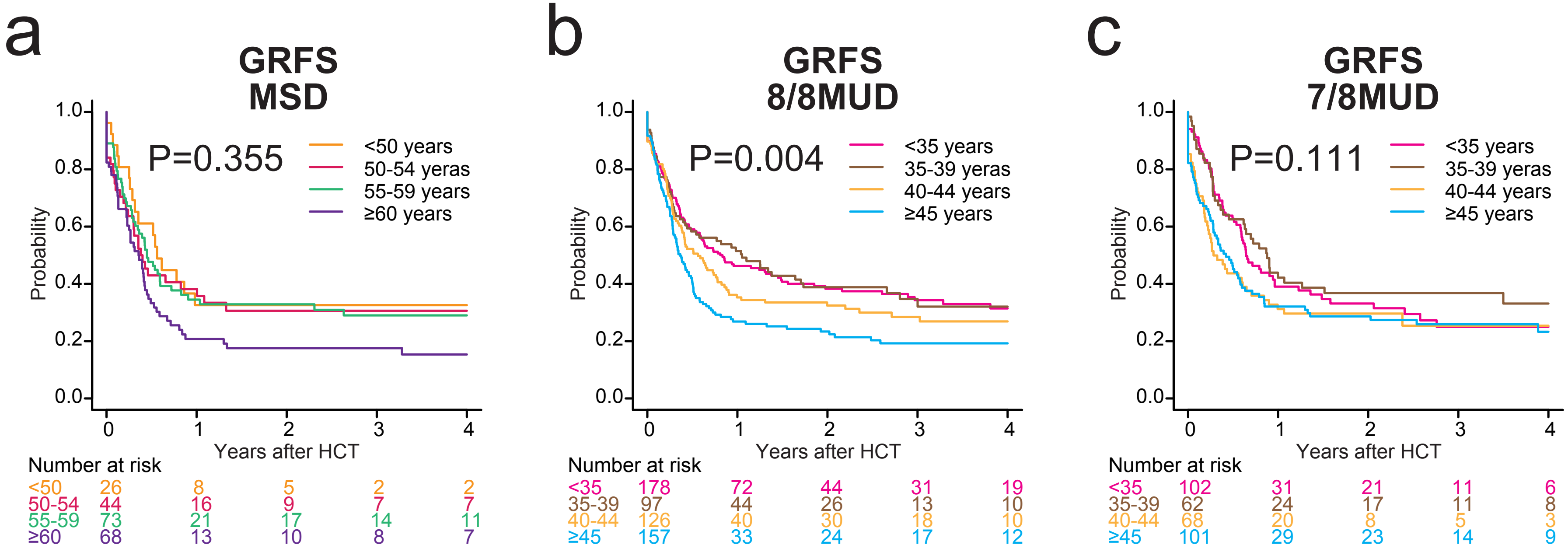

Supplementary Table 1. Multivariate analysis of OS.

		**MUD 30 years**		**MUD 35 years**		**MUD 40 years**		**MUD 45 years**	
		HR (95%CI)	P	HR (95%CI)	P	HR (95%CI)	P	HR (95%CI)	P
**Donor**									
	MSD	Reference		Reference		Reference		Reference	
	8/8MUD with younger donor	0.71 (0.49-1.03)	0.075	0.80 (0.60-1.08)	0.152	0.85 (0.65-1.11)	0.246	0.84 (0.65-1.08)	0.179
	8/8MUD with older donor	0.94 (0.74-1.20)	0.659	0.95 (0.74-1.23)	0.739	0.96 (0.74-1.26)	0.800	1.10 (0.81-1.49)	0.539
	7/8MUD with younger donor	1.35 (0.92-1.99)	0.120	1.19 (0.85-1.65)	0.300	0.98 (0.73-1.33)	0.924	1.05 (0.80-1.38)	0.695
	7/8MUD with older donor	0.97 (0.73-1.27)	0.828	0.96 (0.72-1.29)	0.833	1.11 (0.81-1.51)	0.494	1.00 (0.68-1.48)	0.973
**Recipient age**									
	50-59 years	Reference		Reference		Reference		Reference	
	≥ 60 years	1.18 (0.97-1.42)	0.081	1.18 (0.97-1.42)	0.082	1.16 (0.96-1.40)	0.108	1.16 (0.96-1.40)	0.113
**Recipient sex**									
	Female	Reference		Reference		Reference		Reference	
	Male	1.20 (0.98-1.47)	0.071	1.20 (0.98-1.47)	0.070	1.20 (0.98-1.47)	0.068	1.19 (0.97-1.46)	0.091
**PS**									
	0-1	Reference		Reference		Reference		Reference	
	2-4	1.85 (1.35-2.53)	**<0.001**	1.87 (1.36-2.57)	**<0.001**	1.85 (1.35-2.54)	**<0.001**	1.83 (1.33-2.51)	**<0.001**
**HCT-CI**									
	0-2	Reference		Reference		Reference		Reference	
	≥3	1.45 (1.19-1.76)	**<0.001**	1.44 (1.19-1.76)	**<0.001**	1.43 (1.18-1.75)	**<0.001**	1.43 (1.17-1.74)	**<0.001**
**Karyotype**									
	Others	Reference		Reference		Reference		Reference	
	Poor	1.60 (1.34-1.90)	**<0.001**	1.60 (1.34-1.91)	**<0.001**	1.59 (1.33-1.90)	**<0.001**	1.60 (1.34-1.91)	**<0.001**
**Disease risk at HCT**									
	Low-risk	Reference		Reference		Reference		Reference	
	High-risk	0.99 (0.82-1.19)	0.948	0.99 (0.82-1.20)	0.965	1.00 (0.83-1.21)	0.936	1.00 (0.83-1.21)	0.961
**Conditioning regimen**									
	MAC	Reference		Reference		Reference		Reference	
	RIC	0.83 (0.69-1.00)	0.060	0.82 (0.68-0.99)	**0.046**	0.84 (0.70-1.01)	0.069	0.85 (0.71-1.02)	0.094
**GVHD prophylaxis**									
	With MTX	Reference		Reference		Reference		Reference	
	Without MTX	0.70 (0.48-1.04)	0.082	0.70 (0.48-1.04)	0.083	0.70 (0.47-1.04)	0.078	0.72 (0.48-1.06)	0.101
**Use of ATG**									
	No	Reference		Reference		Reference		Reference	
	Yes	0.63 (0.47-0.85)	**0.002**	0.63 (0.47-0.85)	**0.002**	0.63 (0.47-0.85)	**0.002**	0.63 (0.47-0.85)	**0.002**

OS, overall survival; MSD, matched sibling donor; MUD, matched unrelated donor; PS, performance status; HCT-CI, hematopoietic cell transplantation-specific comorbidity index; HCT, hematopoietic cell transplantation; MAC, myeloablative conditioning; RIC, reduced-intensity conditioning; GVHD, graft-versus-host disease; MTX, methotrexate; ATG, antithymocyte globulin; HR, hazard ratio; CI, confidence interval.

The P values in bold are statistically significant (<0.05).

Supplementary Table 2. Multivariate analysis of GRFS.

		MUD 30 years		MUD 35 years		MUD 40 years		MUD 45 years	
		HR (95%CI)	P	HR (95%CI)	P	HR (95%CI)	P	HR (95%CI)	P
**Donor**									
	MSD	Reference		Reference		Reference		Reference	
	8/8MUD with younger donor	0.65 (0.48-0.89)	**0.007**	0.69 (0.54-0.89)	**0.004**	0.73 (0.59-0.92)	**0.007**	0.77 (0.63-0.95)	**0.016**
	8/8MUD with older donor	0.89 (0.73-1.09)	0.289	0.94 (0.76-1.15)	0.564	0.99 (0.79-1.24)	0.976	1.12 (0.86-1.45)	0.376
	7/8MUD with younger donor	1.07 (0.77-1.50)	0.664	0.93 (0.70-1.24)	0.657	0.84 (0.65-1.09)	0.196	0.95 (0.76-1.20)	0.699
	7/8MUD with older donor	0.92 (0.74-1.16)	0.530	0.97 (0.76-1.23)	0.804	1.11 (0.86-1.44)	0.401	0.99 (0.72-1.36)	0.967
**Recipient age**									
	50-59 years	Reference		Reference		Reference		Reference	
	≥ 60 years	1.15 (0.98-1.34)	0.077	1.15 (0.98-1.34)	0.078	1.14 (0.98-1.33)	0.087	1.13 (0.96-1.32)	0.118
**Recipient sex**									
	Female	Reference		Reference		Reference		Reference	
	Male	1.14 (0.96-1.34)	0.118	1.13 (0.96-1.34)	0.122	1.13 (0.96-1.34)	0.132	1.12 (0.95-1.32)	0.172
**PS**									
	0-1	Reference		Reference		Reference		Reference	
	2-4	1.54 (1.16-2.05)	**0.002**	1.57 (1.18-2.09)	**0.001**	1.59 (1.20-2.11)	**0.001**	1.58 (1.18-2.10)	**0.001**
**HCT-CI**									
	0-2	Reference		Reference		Reference		Reference	
	≥3	1.10 (0.93-1.31)	0.247	1.10 (0.92-1.30)	0.265	1.08 (0.91-1.28)	0.373	1.08 (0.91-1.28)	0.362
**Karyotype**									
	Others	Reference		Reference		Reference		Reference	
	Poor	1.43 (1.23-1.65)	**<0.001**	1.43 (1.24-1.66)	**<0.001**	1.42 (1.23-1.65)	**<0.001**	1.44 (1.24-1.67)	**<0.001**
**Disease risk at HCT**									
	Low-risk	Reference		Reference		Reference		Reference	
	High-risk	1.01 (0.86-1.18)	0.852	1.01 (0.86-1.18)	0.856	1.02 (0.87-1.20)	0.749	1.01 (0.86-1.19)	0.820
**Conditioning regimen**									
	MAC	Reference		Reference		Reference		Reference	
	RIC	0.80 (0.69-0.94)	**0.006**	0.80 (0.68-0.93)	**0.005**	0.80 (0.69-0.94)	**0.007**	0.81 (0.69-0.95)	**0.010**
**GVHD prophylaxis**									
	With MTX	Reference		Reference		Reference		Reference	
	Without MTX	0.91 (0.68-1.22)	0.544	0.91 (0.68-1.23)	0.560	0.89 (0.66-1.20)	0.463	0.92 (0.68-1.23)	0.586
**Use of ATG**									
	No	Reference		Reference		Reference		Reference	
	Yes	0.57 (0.45-0.73)	**<0.001**	0.57 (0.44-0.73)	**<0.001**	0.57 (0.44-0.73)	**<0.001**	0.57 (0.44-0.72)	**<0.001**

GRFS, graft-versus-host disease (GVHD)-free, relapse-free survival; MSD, matched sibling donor; MUD, matched unrelated donor; PS, performance status; HCT-CI, hematopoietic cell transplantation-specific comorbidity index; HCT, hematopoietic cell transplantation; MAC, myeloablative conditioning; RIC, reduced-intensity conditioning; GVHD, graft-versus-host disease; MTX, methotrexate; ATG, antithymocyte globulin; HR, hazard ratio; CI, confidence interval.

The P values in bold are statistically significant (<0.05).
